# An Overview of Severe Myalgic Encephalomyelitis

**DOI:** 10.3390/jcm15020805

**Published:** 2026-01-19

**Authors:** Mark Vink, Alexandra Vink-Niese

**Affiliations:** 1 Independent Researcher, 1096 HZ Amsterdam, The Netherlands; 2 Independent Researcher, 30159 Hannover, Germany

**Keywords:** CFS, chronic fatigue syndrome, functional gastrointestinal disease, gastroparesis, medical gaslighting, ME/CFS, POTS, severely affected (housebound) patients, (very) severe chronic disease

## Abstract

In this article, we have reviewed the literature on severe myalgic encephalomyelitis/chronic fatigue syndrome (ME/CFS). ME/CFS is a clinical diagnosis in the absence of a diagnostic test. However, in research settings and disability disputes, 2-day cardiopulmonary exercise testing can be used to diagnose and document the abnormal response to exercise. Biomedical research into this disease has been scarce and underfunded for decades. Consequently, there are no effective treatments. In its most severe form, it is more disabling than many other diseases, and patients are bedbound 24/7, dependent on carers, and spend their days in dark and quiet rooms. Even the soft sound of a human voice can lead to further deterioration. Some of the very severely ill suffer from life-threatening malnutrition and need to be tube-fed. The COVID-19 pandemic has led to a sharp increase in the number of patients with post-infectious diseases, and many of them fulfill ME/CFS criteria. Dedicated, focused research using advanced medical technologies is needed to gain further understanding of the underlying disease mechanism. This will enable us to find effective pharmacological treatments and address the unmet medical needs of these very ill people.

## 1. Introduction

Myalgic encephalomyelitis/chronic fatigue syndrome (ME/CFS) is a complex multisystem physical disease that affects the endocrine, immune, energy production, and neurological pathways in the body [1]. ME/CFS is characterized by post-exertional malaise (PEM) [1,2], a dramatic effect of exercise upon muscle function in the form of muscle fatigue, muscle weakness, and myalgia; a diurnal variability of symptoms; a multisystem involvement; and a prolonged relapsing course [3].

Patients can have a whole range of other potential symptoms, which can be made worse by mental or physical exertion. These include reversal of the sleep rhythm or other disruptions of the sleep pattern, cognitive dysfunction, dizziness, tinnitus, headaches and migraines, hypersensitivity to light and/or sound, gastrointestinal disturbances, and/or orthostatic intolerance [3]. The disease has been classified as a neurological disease by the World Health Organization since 1969 [4], yet for many years it has been characterized as a psychosomatic disorder by the medical profession. Over the last few decades, most research money has been spent on psychological interventions and on psychologizing the disease. There are no approved effective pharmacological treatments, and the treatments that have been labeled as safe and effective for many years have been found not to lead to improvement or recovery, according to, for example, the British National Institute for Health and Care Excellence (NICE) in October 2021 [2]. Before the COVID-19 pandemic, there were an estimated 250,000 ME/CFS patients in the UK, 40,000–150,000 patients in the Netherlands, 140,000–310,000 in Germany, and around 1.5 million in the US [1,5,6,7,8]. Around 75% of patients are women, and the disease usually follows a viral infection [1,2]. The SARS-CoV-2 pandemic has led to a sharp increase in patients with post-infectious diseases. According to one report, there are as many as 400 million long COVID patients worldwide [9]. Many patients with post-COVID-19 syndrome, or long COVID, suffer from PEM and fulfill ME/CFS criteria. Many ME/CFS experts think that long COVID is ME/CFS triggered by COVID-19, or it has a striking resemblance to that [10]. As a consequence of that, there are now an estimated 650,000 ME/CFS patients in Germany, and according to the same report, long COVID and ME/CFS are costing the German economy EUR 63 billion per year [11].

In an international survey of 1418 people (mean age of 45.8 years, 85.6% were women) with a reported diagnosis of ME/CFS by a health professional, patients were most affected by their inability to perform usual activities, pain, self-care, and mobility, and they were the least impacted by anxiety [12].

A large proportion of patients are too ill to work, and around 25% of ME/CFS patients are severely or very severely affected, and many of them are dependent on care by others [2]. Around 49% of patients suffer from gastrointestinal problems, “nausea/disturbance of intestinal motility” (p. 528, [3]), which in the severely ill can lead to life-threatening malnutrition. Just like in many other chronic diseases, only about 0.5% of the literature focuses on the severely affected. In ME/CFS, this is more of a problem than in other diseases because research into this disabling disease has been underfinanced for decades, so that the number of studies into this disease has been much less compared to other diseases [13,14]. The reason for this, as noted by the Institute of Medicine, is likely to be that many medical professionals still do not believe in the disease or view it as nothing more than being a bit tired. The consequence of this is that ME/CFS is mainly studied in psychiatry and psychology, which further reinforces the incorrect assumption that it is a psychological or psychosomatic disorder [1].

## 2. Methods

The structure of the paper is as follows. We begin by discussing a case report of a 38-year-old woman with severe ME/CFS and addressing important issues that are raised by this case. Following this, we overview the state of knowledge of ME/CFS by using the literature to address important issues like how to diagnose ME/CFS, what the categories of severity are, what severe ME looks like, what the therapeutic options are, and what the prognosis is. A comprehensive search of the literature was undertaken using electronic databases (PubMed, Medline, Google Scholar, Academia, PsycINFO, Scopus, and the Web of Science) for articles on the natural history of ME/CFS, on (very) severe ME/CFS, and on treatments for it in studies that have been published before November 2025. Studies were included irrespective of study designs and irrespective of the diagnostic criteria that were used by those studies. We also searched the reference lists of the articles identified for the review. However, we would like to stress that this is an overview of the literature on severe ME and not a systematic review or a meta-analysis.

Case reports are the lowest in the hierarchy of evidence, with a meta-analysis representing the highest level. Even though case reports are a form of anecdotal evidence, they are important with regard to rare events and grabbing the attention of medical colleagues by presenting interesting cases, cases with challenging diagnoses, unusual presentations, or medical dilemmas, which might be the exceptions to the cases covered by guidelines, so that a pragmatic instead of a dogmatic approach is needed [15]. Moreover, as noted by Råbu and Binder, “case studies can be beneficial for investigating exceptions to general trends and giving voice to underrepresented experiences”. But also, “case studies can be useful to integrate theoretical knowledge with practical application and thereby contribute to development of professional expertise, including practical wisdom and clinical flexibility” (p. 119, [16]).

We will start by discussing the case of Paula, which is not her real name, by the way, to illustrate problems encountered by people with severe ME/CFS. Paula is a Dutch female patient in her late 30s who has been ill for more than a decade after contracting an infection, which triggered her ME/CFS. She is suffering from severe ME, very severe POTS, and gastroparesis. Because of this, she was having gradually worsening nutritional problems for which she was treated with a nasogastric (NG) tube. Because of her gastroparesis, the NG-tube was later placed in her duodenum. Despite that, her weight continued to drop. She lost >20 kg in 1 1/2 years and saw her BMI drop to 16. According to her doctors, intestinal failure (IF) was ruled out with 100% certainty, and therefore, she was diagnosed with functional gastrointestinal disease (FGID). NG-tube feeding was continued despite the fact that it did not help. At the same time, she was started on an antidepressant (escitalopram) and psychoeducation to explain the psychological underpinnings of her nutritional problems to her [17], but she had to stop the escitalopram after a month because of worsening sickness, pain, and other problems. Her doctors wanted her to see a psychiatrist for her FGID, but she did not want that. She did see a psychologist, however, who ruled out mental health problems as the reasons for her malnutrition [18].

The doctors and hospital did not want to treat her with parenteral nutrition (PN), citing an important gastroenterological position paper [19], which is based on the consensus opinion that PN should not be prescribed for patients without IF if the oral and/or enteral route can be utilized. They did not mention, however, that one of them was a co-author of that article. Paula already had a PICC line for her fluids, but they also did not want to start her on PN for a very short, time-limited period, either because of the alleged functional nature of the malnourishment [17].

## 3. Analysis

### 3.1. Reasons for Not Using Parenteral Nutrition (PN)

The authors of the consensus article gave two reasons for not wanting to use PN. First of all, because using PN can lead to catheter-related complications such as thrombosis, bloodstream infections, hepatic, metabolic, renal, and bone complications. Secondly, and even more importantly, because, according to them, the life expectancy is >17 years less for people on long-term PN than for the general population [19].

#### 3.1.1. Complications of PN

The authors of the consensus article note that the complications from long-term use of PN “often require recurrent hospitalizations and will lead to significant morbidity and sometimes mortality” (p. 2280, [19]). A review by Dibb et al., on the other hand, concluded that “home parenteral nutrition delivered by skilled nutrition teams has low incidences of catheter-related complications” (p. 587, [20]).

#### 3.1.2. Life Expectancy

The claim by the consensus document that the average life years lost because of home parenteral nutrition (HPN) is >17 years compared to the general population [19] is based on a large study (*n* = 1046) by Kopczynska et al. [21] spanning more than four decades. This study excluded the 206 cancer patients from its analysis. Analyzing the results from the remaining 840 patients showed that “40 (9.0%) deaths were HPN or IF-related, while underlying disease leading to IF accounted for 98 (22.2%) deaths. There were 270 (61.1%) deaths not related to IF, with the majority of these patients dying from infections unrelated to HPN” (p. 2446, [19]). The consequence of this is that only 9% of deaths were related to HPN.

Dutch women have a life expectancy of 83.4 years [22]. The life expectancy for Paula on HPN would then be around 66 years (83.4—more than 17). Her life expectancy, on the other hand, without any nutrition or HPN, is maximal around two months [23]. Put differently, without HPN, she would die at age 38. If the doctors would treat her with HPN, then her life expectancy would be the aforementioned 66, signifying a dramatic increase of 28 years. This also means that the study by Kopczynska et al. [21] does not provide any evidence that HPN should not be prescribed for patients without IF, contrary to what is claimed by the consensus document [19]. Instead, the study by Kopczynska et al. provides evidence for the opposite.

This same study also shows that there has been a gradual increase in the use of HPN in recent times to improve the quality of life and support surgery or chemotherapy for cancer patients *who do not suffer from IF*. According to that study, 20% (206/1046) of patients who are currently treated with HPN do not have IF.

### 3.2. Dutch Malnutrition Guideline

According to the Dutch malnutrition guideline from 2019, PN is indicated when sufficient nutrition via the gastrointestinal tract cannot be provided for more than seven days because enteral nutrition is not possible, insufficient, or is contraindicated (in Dutch: “Totale parenterale voeding (TPV) is geïndiceerd wanneer er langer dan zeven dagen niet voldoende gevoed kan worden via het maag-darmkanaal omdat enterale voeding niet of onvoldoende mogelijk is of contra-geïndiceerd is” (p. 29, [24])). It is unclear why this is ignored by the Dutch hospitals.

### 3.3. Situation in Germany

The situation in Germany, which is Holland’s neighboring country to the east, on the other hand, is totally different. Here, PN can be used in many cases, for example, if patients suffer from gastrointestinal dysfunction, malabsorption, or maldigestion, or they have a significantly increased energy and nutrient requirement that cannot be met through oral intake alone. Additionally, if patients become malnourished or are at risk of it as a result of chronic or acute illnesses, infection, or surgery, then they can be helped too [25]. The team helping Paula, which consisted of friends, family, her carer, a solicitor, and a few doctors, was able to find her a German hospital where they were willing to treat her with PN. Why they use a patient-centered approach in Germany when it comes to malnutrition and a doctor-centered approach in the Netherlands is unclear.

### 3.4. ME/CFS and Malnutrition

Baxter et al. [26] presented five cases of patients in their article entitled *Life-Threatening Malnutrition in Very Severe ME/CFS*, who were wrongly diagnosed with a mental health disorder as the cause of their nutritional problem. In four of those cases, patients were misdiagnosed with anorexia nervosa, highlighting the lack of knowledge about ME/CFS but also about anorexia nervosa. One can easily tell the difference between severe malnutrition caused by ME/CFS or anorexia nervosa if one talks to the patient for half a minute. ME/CFS patients are not happy at all to be malnourished, and they are very keen to receive appropriate help to put on weight. Anorexia nervosa patients, on the other hand, have a distorted self-image, and they see themselves as overweight, whereas everybody else can see that they are malnourished and very thin [26]. Why doctors do not know this simple difference is unclear, because knowing what anorexia nervosa is is basic psychiatric knowledge that all doctors should have. Knowing this difference is even more important because the delay in instigating tube feeding led to severe and life-threatening malnutrition in all four patients.

Most doctors are not aware of the fact that nutritional difficulties are relatively common and a direct consequence of (very or extremely) severe ME/CFS. There are a number of reasons for these nutritional difficulties. The most common one is that the sheer effort of eating and drinking is too much for patients. Other possible reasons are swallowing difficulties and problems such as mast cell activation disorder, gastroparesis, autonomic dysfunction, or features of malabsorption. Another lesser-known reason is the extreme sensory sensitivity to sound in very severe or extremely severe ME/CFS. The consequence of this is that chewing by the patient itself might be too noisy, leading to further deterioration, even if the patient is not eating noisy food like paprika, raw carrots, et cetera. A way around it might be pureeing food or eating liquid foods like soup or liquid dinners, which one can easily buy in the supermarkets these days. However, even in this case, swallowing can be too noisy for some patients, which can lead to further deterioration. It might well be that because of this, these patients also need tube feeding. In some cases of nutritional problems in ME/CFS, enteral tube feeding fails, and PN is necessary.

When ME/CFS is very or extremely severe, it might lead to impaired function of the esophagus, stomach, and intestines because these processes, which are controlled by the autonomic nervous system, are frequently disrupted in ME/CFS. This can significantly hinder or prevent food intake and can lead to severe malnutrition (in German: “Beeinträchtigte Funktion von Speiseröhre, Magen und Darm: Diese durch das autonome Nervensystem gesteuerten Prozesse sind bei ME/CFS häufig gestört. Das kann die Nahrungs- aufnahme erheblich erschweren oder unmöglich machen” (p. 5, [27]).

The risk of refeeding syndrome is increased by misdiagnosing a severe physical disease as a mental health problem and the lack of timely and appropriate action by healthcare professionals due to a lack of knowledge about ME/CFS [26]. But also about POTS because that can also lead to severe nutritional difficulties. Refeeding syndrome is characterized by a dangerous shift in fluids and electrolytes, including phosphorus, potassium, and magnesium, within the body and occurs in a small subset of severely malnourished patients when calories are introduced too quickly, lab tests are not checked, and abnormalities are not treated [28]. Common signs of this syndrome are low levels of phosphorus in the blood and edema, or swelling in the lower extremities. It usually occurs in the first five days of introducing and increasing food again [28]. The syndrome can be life-threatening, leading to complications such as arrhythmia, cardiac failure, cardiac arrest, delirium, and seizures [29].

### 3.5. Similar Case

A number of years ago there was a similar case in the UK involving Maeve Boothby O’Neill. The medical professionals involved in the care of that extremely severely affected patient did not believe in ME/CFS, nor were they aware of how severe it can be. Just like most doctors are not aware of the fact that the World Health Organization has classified ME/CFS as a neurological disease since 1969. Or that the British NICE Institute concluded in its 2021 ME/CFS guideline that people with very severe ME/CFS “need help with personal hygiene and eating, and are very sensitive to sensory stimuli. Some…may not be able to swallow and may need to be tube fed” (p. 9, [2]). But also that these patients are bedridden 24/7 and totally dependent on care. Maeve’s life-threatening malnutrition was not adequately treated as a consequence of the medical disbelief, which resulted in her death. This could have easily been prevented if the knowledge of the medical professionals involved had been up-to-date. Shortly before she died, Maeve said: “this is a f***ing horrible way to die” [30].

### 3.6. POTS and Malnutrition

Gastrointestinal (GI) dysmotility and a variety of severe GI symptoms are common in POTS [31]. The most common GI abnormality in POTS is delayed gastric emptying [32]. A review by Kornum et al. [33] concluded that motility tests, such as those used by the Dutch hospitals, are often falsely negative in POTS patients: “some patients may have enteric neuropathy despite normal motility measurements” (p. 3). Also, “evidence suggests multi-segmental dysmotility in the GI system of both patients with POTS and DM” (p. 8). They finished by concluding that “with no available standard diagnostic test of pan-enteric autonomic neuropathy, extraintestinal autonomic neuropathy may be used as a proxy in clinical practice to verify AD [autonomic dysfunction] outside the GI tract” (p. 16, [33]). The gold standard to diagnose POTS is the tilt table test.

A study by van Campen et al. [34], involving 429 patients with mild and moderate ME/CFS, found that 86% had orthostatic intolerance symptoms during daily life. All of these patients underwent the tilt table test, which showed that 90% had an abnormal cerebral blood flow (CBF) reduction as assessed by extracranial Doppler measurements. Interestingly enough, this was not only present in ME/CFS patients with POTS and delayed orthostatic hypotension, but also in those patients who had a normal heart rate and blood pressure response during tilt table testing. Another important conclusion from this study is that the mean CBF reduction (26%) in the 429 patients with ME/CFS was significantly different from the 7% reduction observed in healthy controls. It is also important to note that tilt table testing can induce PEM, and in some patients, it even leads to flare-ups and relapses. Moreover, patients with a previous diagnosis of POTS had a larger CBF reduction compared to patients without POTS. The (very) severely ill are (often) too ill for tilt table testing. However, research involving 100 severely ill patients who were compared to healthy controls suggests that a sitting test is an adequate alternative for diagnosing orthostatic intolerance in the severely affected [35].

Tilt table testing showed that Paula had a 45% reduction in blood flow to her head, which shows that she has very severe POTS. If the GI tract were affected in a similar manner, then life-threatening malnutrition could be a logical consequence of that.

### 3.7. Gastroparesis and Malnutrition

According to Bharadwaj et al. [36], the majority of gastroparesis (GP) patients are at risk of significant nutritional abnormalities. Also, “a systematic approach including initial nutritional screening, diet recommendations, … and enteral and parental nutrition should be considered in complex GP patients” (p. 285), like Paula, who was therefore referred from her regional hospital to two university hospitals, but both refused to treat her with PN, as discussed earlier. The third of three university hospitals that can treat patients with PN in the Netherlands did not want to help her either, citing her postcode, which shows that she lives on the other side of the country, as the reason to refuse to do so.

### 3.8. Visceral Hypersensitivity

The doctors also use a Dutch article by Korzilius et al., co-authored by two of them, in which they state that patients are increasingly urging their healthcare providers to initiate PN without a clear medical indication [37]. They argue that this should not happen in the absence of IF. Yet they also note that patients with normal gastrointestinal motor function may still have difficulty tolerating food if they have visceral hypersensitivity (VH), an eating disorder, or an affective disorder (p. 3, [37]). Steinsvik et al. [38] found signs of VH using an ultrasound drink test in ME/CFS patients with abdominal complaints. VH is also linked to autonomic dysfunction (AD) in a significant number of POTS patients [33]. Korzilius et al. also state that these patients are usually young women with psychotrauma in the past who refuse to see a psychiatrist, who have frequent contact with similar patients, and who have frequent infections of the PN catheter with unusual bacteria [37]. Thereby suggesting that these patients have a psychological and not a physical problem.

### 3.9. Disbelief by the Medical Professional Leading to Misclassification as Psychogenic

Patients with conditions such as ME/CFS, fibromyalgia, Lyme disease, and POTS “are often told their symptoms are imaginary or selfinflicted” (p. 03, [39]). But as noted by Monaco et al. [39], misclassification of these conditions as psychosomatic or psychogenic leads to stigmatization and delayed care. Psychiatrists Klaassen and VanDerNagel [40] concluded something similar about POTS. This misclassification often leads to avoidable life-threatening malnutrition.

Disbelief on the part of the medical profession about the nature of the disease is a direct result of the claim by proponents of the cognitive behavioral model that there is no underlying disease. This disbelief is not only reserved for the mildly affected, but the severely affected are not protected from this either. According to Speight, there are a number of levels of disbelief [41]. The first one is doctors who do not believe that it is a physical disease and who are not aware of the aforementioned classification by the World Health Organization. The second form of disbelief is that doctors believe in the disease, but they do not believe that a particular patient has the disease. One step up from this is that many doctors still believe in the efficacy of Cognitive Behavioral Therapy (CBT) and/or Graded Exercise Therapy (GET), even though this has been refuted after an extensive review of the literature, by, for example, the British NICE Institute [2,42]. Convinced of the efficacy of these treatments, doctors then change the diagnosis when patients do not respond to them, unaware of the fact that those treatments do not lead to improvement or recovery. Nor do they lead to objective improvement. Even the British Association of Clinicians in ME/CFS (BACME), which is run by proponents and supporters of these 2 treatments, has now concluded in its ME/CFS Guide to Therapy 2025, that “GET is NOT supported by this updated NICE Guideline”. Because it is based on “fixed increments” of increase and it does not allow “people to step back if symptoms worsen” (p. 27 [43]). The final level of disbelief is when the doctor changes the diagnosis when it progresses from mild or moderate to severe because he does not believe that the disease can become that bad. Instead, the diagnosis is then changed to a psychological one. And just like at any of the other levels of disbelief, doctors often involve psychiatrists to relabel physical problems as mental health problems. If patients are still underage, then this might lead to unwarranted claims that patients suffer from Pervasive Refusal Syndrome or that the parents are making and/or keeping the child ill. In both cases, this can lead to health professionals traumatizing the child by involving social services or even removing the child from the care of his or her parents under the false pretenses of Munchhausen by proxy or child abuse [44].

### 3.10. Antidepressants for Functional Gastrointestinal Disorders

Gastrointestinal symptoms like pain, dyspepsia, and altered bowel habit, without an organic explanation, are highly prevalent. According to Fikree et al. and Black et al., common FGIDs are irritable bowel syndrome, functional dyspepsia, or constipation, which are made worse by maladaptive patient behaviors [45,46]. Many physicians view them as psychiatric instead of real physical diseases, leading to negative attitudes towards these patients. This is reinforced by the fact that selective serotonin reuptake inhibitors (SSRIs) and tricyclic antidepressants (TCAs) are used to treat FGID, as we have seen in Paula, who was treated with escitalopram, which is an SSRI [47]. Even though the evidence supporting the use of antidepressants in managing FGID is weak [48,49]. TCAs are not effective in the acute phase in malnourished anorexics [49,50]. There is no evidence supporting the use of SSRIs and TCA in patients suffering from severe or life-threatening malnutrition caused by other conditions, either [49]. Also, antidepressants are not indicated as treatment for ME/CFS [42]. Moreover, TCAs can cause rapid weight gain, which might increase the risk of developing the refeeding syndrome. SSRIs can cause QT interval prolongation, which can cause ventricular arrhythmia and can result in sudden cardiac death. Additionally, SSRIs had no effects on symptoms of malnourished underweight anorexics [50]. Consequently, using SSRIs and TCA in patients suffering from severe or life-threatening malnutrition in FGID, or for that matter, severe ME/CFS and very severe POTS, is a form of opinion instead of evidence-based medicine.

## 4. Diagnosis

### 4.1. Diagnostic Test and Post-Exertional Malaise (PEM)

Many doctors think that it is difficult to diagnose ME/CFS because of unfamiliarity with the disease and because there is no diagnostic test for ME/CFS. According to patient surveys, in up to 77%, it took longer than 1 year to obtain a diagnosis, and in almost 30%, it took longer than 5 years [1]. An important reason for this is that most medical schools do not include ME/CFS in their curriculum [51], just like most medical textbooks do not include information on this condition, and if they do, then it is usually incorrect and based on the notion that it is a psychosomatic disease that can be treated and cured by psychological interventions [52].

It is correct to say that there is no blood test to diagnose ME/CFS, but it is incorrect to say that there is no diagnostic test for it. Just like it is incorrect to state that there is no underlying disease just because routine laboratory and other routine tests, which are performed, are normal, which is what insurance doctors have been saying for a long time in the Netherlands. That is, until a recent ruling from the highest disability court in the Netherlands [53].

A study by Beentjes et al. [54] that used UK Biobank data of 1455 ME/CFS cases and 131,303 controls, showed that the levels of 511 blood-based biomarkers differed significantly between people with and those without ME/CFS. The authors concluded that the large number of replicated and diverse blood biomarkers that differentiate between ME/CFS cases and controls should now dispel the myth that ME/CFS is a psychological or psychosomatic disorder.

It has been known for a long time that 2-day cardiopulmonary exercise testing (2-day CPET), according to the protocol from the Workwell Foundation [55], can demonstrate the abnormal recovery to exertion in an objective manner on the second day of that test. Two-day testing is essential to reveal post-exertional malaise (PEM), the main characteristic of ME/CFS, which is also a core dysfunction seen in many long COVID patients [56]. A study by van Campen et al. that compared three groups of ME/CFS patients with each other (mild, moderate, and severe cases) showed that the deterioration in peak workload from day 1 to day 2 is largest in the severe ME/CFS patient group [57]. Of note is that very severely and extremely severely affected patients are too ill to undertake CPET.

A study by Jothi et al. that used invasive CPET (iCPET), which is CPET with a pulmonary artery catheter and arterial line, found an impaired skeletal muscle oxygen diffusion in ME/CFS and long COVID when compared to healthy controls, but their findings “are limited by small sample size” (p. 1, [58]) with only 15 long COVID patients, 11 ME/CFS patients, and 11 controls.

However, a much larger study by Joseph et al. used iCPET to compare 160 ME/CFS patients to healthy controls. Their conclusion was that “the results identify two types of peripheral neurovascular dysregulation that are biologically plausible contributors to ME/CFS exertional intolerance—depressed Qc [cardiac output] from impaired venous return, and impaired peripheral oxygen extraction” (p. 642, [59]).

Additionally, a very large study by Squires et al. used iCPET to compare 438 ME/CFS patients with 73 long COVID (LC) patients and 43 controls. The authors concluded that “ME/CFS and LC share symptomatic, reduced aerobic capacity at peak exercise, which is driven by preload insufficiency and impaired systemic O2 extraction, the latter compatible with peripheral left-to-right shunting and/or limb skeletal muscle dysfunction” (p. 1, [60]).

Due to the exertional nature and intensity of the 2-day CPET, ME/CFS patients whose disease is characterized by PEM will, on average, take two weeks to recover from something that only takes one or two days for healthy people [55]. The problem with this test; however, is that it might also lead to a prolonged flare-up or even to a (major) relapse. For this reason, guidelines caution against routinely using the test in clinical care and only using it in research settings or for settling disability disputes, but only when the potential benefits outweigh the risks [61]. The consequence of this is that the diagnosis of ME/CFS in routine medical care is presently a clinical diagnosis based almost entirely on a carefully taken history and using diagnostic criteria, of which there are a number of different ones. Much of the research has been performed using the Oxford criteria [62], i.e., six months of disabling fatigue, which needs to be present for at least 50% of the time, which does not require the main characteristic of the disease. And the Fukuda criteria, according to which PEM is only an optional requirement [63]. Two of the other problems of the Fukuda criteria are that they are “overly inclusive” and that “many of the minor symptoms overlap with the symptoms of major depression, patients with major depression may be misclassified by the Fukuda definition” as having ME/CFS (p. 48, [1]). Moreover, the Fukuda criteria are polythetic, because two patients could both be diagnosed with ME/CFS despite having very little symptom overlap, which leads to even greater heterogeneity of patients diagnosed this way [1].

The Oxford criteria should not be used anymore because, for example, the American Agency for Healthcare Research and Quality (AHRQ) in 2016 found that there is “a high risk of including patients who may have an alternate fatiguing illness or whose illness resolves spontaneously with time” [64]. The American NIH (National Institute of Health) came to a similar conclusion in 2014 [65,66]. As a matter of fact, a large study by Baraniuk (*n* = 6175) [67] showed that up to 85% of patients diagnosed with the Oxford criteria are, in fact, healthy people. The best criteria currently in use are the international consensus criteria (ICC). According to the ICC, PEM is a compulsory requirement for diagnosis, and patients with ‘mild’ ME/CFS have a 50 percent reduction in pre-illness activity [68]. Also, there is no 6-month waiting period before a diagnosis can be made, contrary to the Fukuda and Oxford criteria. PEM, which the ICC calls post-exertional neuroimmune exhaustion, is compulsory for diagnosis, just as symptoms from three symptom categories (neurological impairments; immune, gastrointestinal, and genitourinary impairments; and energy production impairments) are. The problems of not having a blood test to diagnose ME/CFS were highlighted by Jason et al. [69], who compared a number of different selection criteria. They found that because the criteria do not have a minimum threshold for severity of symptoms, 33.7 percent of healthy controls fulfilled the Fukuda definition, 20.7 percent fulfilled the Canadian consensus criteria (CCC), and 14.6 percent fulfilled the ME-ICC. When this was addressed by adding minimum thresholds, i.e., symptoms must be present at least half of the time with at least moderate severity, then there was a dramatic reduction in the number of healthy controls that fulfilled the criteria (Fukuda: 4.7%, and CCC and ME-ICC: ‘only’ 3.7%).

Many health professionals think that post-exertional fatigue, i.e., tiredness after exercise, is PEM, even though tiredness after exercise is simply a normal physiological response to exercise. Some also claim that worsening of symptoms after exercise is PEM. Yet something similar is seen if people are injured and they return to sports too quickly or do too much too quickly. PEM, however, is something different, and the following four elements are compulsory for a diagnosis of PEM:

- a disproportional worsening of symptoms,
- following trivial physical or mental exertion,
- with loss of strength and/or loss of function,
- and an abnormally delayed recovery [70].

### 4.2. Epidemiological Characteristics

According to a study (*n* = 420) by microbiologist Dowsett, which also involved infectious disease specialist Dr. Ramsay, who documented the outbreak of an unknown disease in the Royal Free Hospital in London in 1955, which later became known as ME [3,71], 81% of patients have an acute onset after a respiratory/gastrointestinal infection or a flu-like illness [3]. The remaining 19% had an insidious onset. 41% of patients in that cohort worked in healthcare or teaching. The average age of onset was 32, and the peak incidence is between 30 and 39. But also between 10 and 19 [1,2,72]. The illness was reported to be improving in 31%, fluctuating in 20%, remaining unchanged in 25%, and becoming worse in 24%. ME/CFS is said to be a relapsing, remitting disease, and known precipitants of relapses included going over one’s limit and physical and mental stress in all 420 patients, infections in 42%, climatic change or hot baths in 12%, surgery, immunization, and hormonal disturbances in 9%, and different forms of medication (psychoactive, antiarthritic, or steroid drugs) in 5% [3].

### 4.3. Alterations in Plasma Immune Signatures

Hornig et al. found “distinct alterations in plasma immune signatures early in the course of ME/CFS (*n* = 52) relative to healthy controls (*n* = 348) that are not present in subjects with longer duration of illness (*n* = 246). Analyses based on disease duration revealed that early ME/CFS cases had a prominent activation of both pro- and anti-inflammatory cytokines as well as dissociation of intercytokine regulatory networks. We found a stronger correlation of cytokine alterations with illness duration than with measures of illness severity, suggesting that the immunopathology of ME/CFS is not static” (p. 1, [73]).

### 4.4. Impaired Mitochondrial Function

Tomas et al. investigated the oxidative phosphorylation and glycolysis in moderately (*n* = 39) and severely affected patients (*n* = 38), but also in healthy controls [74,75]. They found that severely affected patients have both mitochondrial and glycolytic impairments, contrary to the moderately affected patients, who only have mitochondrial impairment, which might explain the difference in severity between the two groups. The study also concluded that mitochondrial function is impaired in ME/CFS irrespective of the severity of the disease.

Charlton et al. demonstrated that ME/CFS as well as long COVID patients suffer from reduced exercise capacity and skeletal muscle abnormalities, which cannot be explained by deconditioning. They also noted that these patients “exhibit lower aerobic exercise capacity and mitochondrial respiration than healthy individuals” (p. 3, [76]). Davenport et al. showed recently that this cannot be explained by deconditioning, effort preferences, or other psychological factors [77].

Moreover, as long ago as 1990, Dowsett et al. concluded that “exercise related abnormalities of function have been demonstrated…including a failure to coordinate oxidative metabolism with anaerobic glycolysis causing abnormally early intracellular acidosis, consistent with the early fatiguability and the slow recovery from exercise in ME” (p. 529, [3]).

### 4.5. Different Abnormalities in Short- and Long-Term Illness

Multi-omics research by Xiong et al. compared patients who had been ill for less than four years (*n* = 75) with those who had been ill for more than 10 years (*n* = 79) to healthy controls (*n* = 79). Their study showed that AI-based analysis of metabolome, immune profile, and microbiome is able to clearly distinguish ME/CFS from depression and fibromyalgia. But also that patients who had been ill for a short period of time “showed significant microbial dysbiosis”, while patients who had been ill for a long period of time, “had largely resolved microbial dysbiosis but had metabolic and clinical aberrations” (p. 2, [78]).

### 4.6. Spectrum of ME/CFS Severity

According to NICE, ME/CFS patients are often neglected, and the severity of their symptoms is misunderstood. According to a number of studies, ME/CFS patients are more functionally impaired than those with other disabling illnesses, including those with five or more chronic health conditions, acute myocardial infarction, cerebral thrombosis, end-stage renal disease, congestive heart failure, angina and rheumatoid arthritis, multiple sclerosis (MS), lung cancer, stroke, or ischemic heart disease [79,80,81,82].

One could argue, as the IOM has [1], that the term *mild ME/CFS* is an oxymoron because *mild* ME/CFS, as defined by the international consensus criteria (ICC) [68], with a substantial (50%) loss of function compared to before the onset of the disease, is also a severe disease.

A study by Conroy et al. [83] found that 25.7% in a study of 2138 participants were severely affected; this is almost the same as the 25% that is normally used for that [2,84]. Extrapolating this to the 1.5 million persons who are estimated to suffer from ME/CFS in the United States means that 385,000 people are homebound due to severe ME/CFS. Of the 2138 participants in the same study [83], 4.2% (89/2138) were found to be bedridden, which equates to more than 16,000 people in the US. This indicates a serious public health problem for 385,000 severely ill people, of whom 16,000 are very severely ill, for whom there is no effective treatment.

According to a survey by the European ME Alliance from 2024 [85], involving 11,109 patients, 53.8% had moderate ME/CFS, 16% had severe ME/CFS, while 2.4% had very severe ME/CFS and were bedbound and in need of care.

However, it is likely that the percentage of very severely affected patients in both studies is substantially higher because these patients are often too ill to take part in these sorts of studies. Consequently, it is likely that at least 20% to 25% of patients are (very) severely affected, and a small percentage of them are extremely severely affected. How small or large that percentage is, is unclear because, as mentioned, research involving these patients does not happen very often [13,84] although according to an article by Abbot, on behalf of ME Research UK and the 25% ME Group, that percentage is often estimated to be 25%, which is why the charity representing and helping patients with (very) severe ME, is called the 25% ME Group. Abbot also noted that a survey by Action for ME in 2000 showed that 34% described themselves as severely affected, and according to the aforementioned Dr. Ramsay, one third experiences “a severe and debilitating downhill course” (p. 5, [13]).

The severity and the level of functional impairment seen in ME/CFS vary from person to person but might also fluctuate a bit. Moreover, the symptoms and severity of it as experienced by a patient may present differing levels of severity. For instance, the physical impairment of arms and legs might not be to the same extent, so that, for example, the impairment of the arms might be more severe than that of the legs or vice versa. The same applies to the level of cognitive impairment and the often accompanying orthostatic intolerance.

A mildly affected adult patient may still be able to work, depending on how physical the work is, and mildly affected children and adolescents might still be able to attend school. One often reads that the most severely affected patient *may be bedbound and may need total care*. In reality, the most severely affected, those who are very severely affected or extremely severely affected, are bedbound 24/7 and unable to care for themselves. Table 1 shows the spectrum of severity. NICE [2] itself comes up with four levels of severity, but we have added a fifth level of severity. This group of people is often referred to as the living death, yet this group of patients is normally not mentioned, which might be due to the fact that these patients are extremely ill, dependent on care around the clock, and therefore there is hardly any study about them because they are unable to go to the doctors, so their suffering takes place away from the eyes of the medical profession. This also means to say that most doctors are not aware of their existence. Patients with very severe and extremely severe ME/CFS are very sensitive to light and sound. The consequence of this is, for example, that exposure to all sorts of normal day-to-day sounds or normal light, for example, daylight or sunlight, which other people do not even notice, can lead to PEM, flare-ups and relapses. These patients lie in dark and quiet rooms, wearing sunglasses or sleeping masks and earplugs or ear defenders to protect themselves from light and sound, to try to protect themselves from further deterioration because even the soft sound of a voice can lead to deterioration.

#### 4.6.1. Most Troublesome Symptoms

According to a small study that compared 20 patients with severe ME to healthy controls, the most troublesome symptoms were fatigue, pain, cognitive impairment, orthostatic intolerance, sleep disturbance, neurosensory disturbance, GI tract impairment, and post-exertional malaise [89]. The severely affected in that study had a physical functioning score of 13, compared to 99 for healthy controls (scale: 0–100, higher scores reflecting better physical functioning). That score would be below 5 and close to 0 for the very severely and the extremely severely affected. In these patients, physical functioning, energy, fatigue, and related functioning were extremely low, but “emotional well-being was clearly less impacted—a clear distinction from the frequent misdiagnosis of clinical depression in these patients” (p. 15, [89]). The authors also noted that there is a striking similarity in symptoms between long COVID and ME/CFS.

Life for those with very or extremely severe ME/CFS may be reduced to basic survival, struggling moment by moment to breathe, eat, and drink, while enduring extreme pain. One of the patients with extremely severe ME was Emily Collingridge, who died of a respiratory arrest on 18 March 2012 after a long struggle with ME/CFS. She said the following about some of her symptoms. “I had no idea that modern medicine could allow such suffering. I lost the ability to speak, to see, to move. I was doubly incontinent, often paralysed, tube fed and in unbelievable pain, only partially relieved by high dose morphine … I could bear no stimulation, even though I couldn’t open my eyes and was in a blacked out bedroom, my eyes had to be covered at all times; I wore earplugs for 23 h per day and someone’s mere presence in my room was like an assault” (p. 3, [90]). This might sound extreme to some, but unfortunately, this is the, to many medical professionals, unknown reality of extremely severe ME/CFS.

#### 4.6.2. Flare-Up

When post-exertional malaise lasts longer than a few days, it is called a flare-up, even though many patients still refer to it as PEM. A flare-up affects the person’s ability to perform their usual activities in a negative way. They are usually triggered by an infection, going over their limits, or other triggers. The worsening of symptoms and loss of function are transient. Flare-ups typically resolve within one or two weeks in response to a temporary drastic change in energy management, but some might take a lot longer to resolve.

#### 4.6.3. Relapse

A relapse is a sustained and marked exacerbation of symptoms lasting longer than a flare-up and needing a substantial and sustained adjustment to what a person is able to do. It may not be clear in the early stages of a symptom exacerbation whether it is PEM, a flare-up, or a relapse. An important difference between PEM, flare-ups, and relapses is that the first two lead to a temporary loss of function, whereas relapses lead to a permanent loss of function, which is disproportionate to the reason for the relapse. A relapse also often leads to new and different symptoms. For example, patients might suddenly become hypersensitive to light and/or sound, which they were not before the relapse. Another characteristic of a relapse is that patients go backwards by 20 steps, and then over the course of weeks or months or more, they go forward by one or two steps. The net effect of a relapse, therefore, is a further major deterioration.

#### 4.6.4. Managing Flare-Ups and Relapses

It is always important to identify the trigger of the flare-up or the relapse to try and see if it is possible that patients are able to avoid that trigger the next time. If the trigger, for example, is an infection, then patients can try to reduce the risk of becoming infections. An easy intervention to try and achieve that with respect to airborne infections is wearing FFP2 face masks if patients are in contact with other people.

As soon as patients realize that there is a flare-up or relapse, the activity level has to be dramatically reduced. The best strategy to stabilize symptoms and recover as soon as possible from a flare-up and to minimize the effect and impact of a relapse is complete rest and a dramatic reduction in activity to almost zero. A period of enforced complete rest at the beginning of a flare-up or relapse might initially be very difficult to do, but it is the only way to recover as soon as possible from a flare-up and to try to minimize the deterioration caused by a relapse.

#### 4.6.5. Vitamin D

A study of 100 patients found that 68% had vitamin D deficiency, 22% had insufficient vitamin D levels (20–30 ng/mL), and only 10% had sufficient levels (>30 ng/mL). The study did not include homebound and bedbound patients, and it is likely that if they had been included, that the percentage of patients with vitamin D deficiency would have been even higher because they are not exposed to sunlight [91]. A similar study, but then involving 350 patients, also found that 68% of participants had vitamin D deficiency [92].

#### 4.6.6. Lifestyle

ME/CFS patients are typically fit and active people who were never ill before they fell ill, usually with a viral infection that triggered their ME/CFS. A large Dutch study (*n* = 247) tested the hypothesis that ME/CFS patients with an unhealthy lifestyle are more functionally impaired compared to patients with a healthy lifestyle. The study found, however, that patients have a healthier lifestyle than the general Dutch population. Significantly fewer CFS patients were overweight, more patients abstained from alcohol, and fewer patients smoked. They also concluded that their hypothesis was incorrect [93].

#### 4.6.7. Comorbidities

People with ME/CFS are often diagnosed with comorbidities. For example, disorders of autonomic function often manifest as POTS, mast cell activation syndrome, fibromyalgia, irritable bowel syndrome, hypermobile Ehlers–Danlos syndrome, hypovitaminosis D, endometriosis, and cardiovascular disease [94,95,96,97,98,99,100]. Two other common comorbidities are depression and anxiety. As noted by Brown et al., 45% to 50% of ME/CFS patients have psychiatric comorbidities, according to a number of studies [101]. Something similar was reported by the PACE trial (*n* = 641) [102], which found that 14% had ME/CFS and anxiety, 14% had ME/CFS and depression, and 18% had ME/CFS and both depression and anxiety disorders. The other 54% did not suffer from a mental health disorder, according to the four authors, who are all mental health professors. However, the study used the Oxford criteria, which can mislabel psychiatric patients as ME/CFS patients. Consequently, the percentage of ME/CFS patients who suffer from anxiety and/or depression is likely to be lower. We could not find a study that directly compares the percentages of mental health problems in ME/CFS with other chronic diseases. Studies that looked for those in other chronic diseases showed, for example, that 41% of patients with hypertension, 45% of patients with type 1 diabetes, and 67% of cancer patients suffered from comorbid depression [103]. A major depressive disorder occurs in 13 to 42% of patients with rheumatoid arthritis [104] and in 23% of inpatients with coronary artery disease (CAD), and a two-year follow-up of CAD patients found that 27% had scores suggestive of depressive symptoms and 41% of anxiety and depression [105]. A study that looked into that in cancer patients found that 38% had symptoms of depression, while 23%, 22%, and 17% had mild, moderate, and severe depression, respectively [106]. Therefore, the percentages of ME/CFS patients with a comorbid mental health disorder are similar to those in other chronic diseases but higher than in healthy people. One of the explanations for that is that chronic diseases have a negative effect on work status, the ability to exercise, family, relationships with higher divorce rates and broken partnerships, loss of friends and social contacts leading to loneliness and abandonment. Moreover, in many patients, it leads to poverty and financial hardship when jobs are lost, with ensuing loss of income [107,108]. So that patients with chronic diseases experience many more negative life events, which are associated with depression [107].

Studies that looked into comorbidities also labeled migraine, insomnia, headaches, and muscle pain as comorbidities [95,98,99]. However, these are not comorbidities but are simply common symptoms of ME/CFS; for example, the M in ME stands for myalgia and is therefore an important symptom and component of the disease and not a comorbidity. The study by Dowsett et al. from 1990 that documented symptoms in 420 patients with ME, found that 80% suffered from myalgia, 76% from headaches, and 64% suffered from reversal of sleep rhythm [3].

### 4.7. It Must Be Psychosomatic

The notion that ME/CFS is a psychosomatic disorder is deeply anchored in the thinking of the medical profession, as highlighted, for example, by a study by Neu et al., which stated that “CFS is a nosographically defined psychosomatic condition” (p. 2, [109]), despite the fact that their study showed “a significant difference of force decrease presents during phasic trials” (p. 10, [109]) with healthy controls when using the hand grip strength test. “In addition, patients showed a significantly different fatigability during phasic trials than HCs” (healthy controls) (p. 9, [109]). Instead of acknowledging this and the fact that they did not have any proof that it is a psychosomatic disorder, but also that their own study contradicted it, they simply stated that “measures of impaired physical force might also be related to motivation in CFS patients” (p. 9, [109]). Blaming the patient is a typical example of medical gaslighting, which is often used by the medical profession if they do not understand a disease or disorder straight away. The consequence of it, however, is that medical professionals are traumatizing patients instead of helping them, which is also known as clinician-associated traumatization. This often stems from the lack of knowledge of medical professionals about certain diseases and their dismissive, disbelieving, and hostile attitude towards patients with those diseases. Many of these patients come to expect clinicians’ negative attitudes and disbelief, the disrespectful and/or unprofessional attitude, and the way they are treated by clinicians who lack humility when they encounter a disorder about which they know little and much less than the patient themselves. This negative attitude by the medical profession seems to be worse in diseases that predominantly affect women, and subsequently, it labels them as psychosomatic disorders. This is a form of misogyny that seems to stem from a low level of trust in women’s abilities to understand their own bodies and the fact that many doctors view complaints they do not understand as irritating and as a sign of hysteria. The way patients experience this is through manipulation tactics such as dismissing, silencing, or blaming symptoms on stress, PMS, or anxiety [110].

#### 4.7.1. Many Diseases in Women Show up Differently

Many doctors do not realize that many diseases in women show up differently than in men because this is something we are not taught in medical school. One of the reasons for this, according to the Association of American Medical Colleges (AAMC), is that before 1993, women were rarely included in clinical trials [111]. The result of this is that women are more likely to have their pain dismissed as emotional or psychogenic and, therefore, are more likely to be given sedatives instead of pain medications. A survey of 12,000 European patients showed that receiving a mental health diagnosis can delay receiving the right diagnosis of a rare disease by 2.5 to 14 times [112]. Moreover, according to Clark, clinicians “with poor communication skills, poor knowledge and unwilling to admit they ‘don’t know’” who are “unwilling to learn anything new…blame the patient for their clinical knowledge insufficiencies, say there is nothing wrong or think the patient is neurotic rather than be prepared to see any deficiency in knowledge within their own clinical skillset” (p. 2, [113]). Many of these doctors behave this way because they are accustomed to never being questioned or held accountable unless someone files a malpractice suit [114]. Every doctor knows that every hoof step is not a zebra, but putting this into practice and diagnosing rare conditions, or conditions they are not familiar with, in a correct way, is challenging, as one of the other things we do not learn in medical school is to say that we don’t know. “Not knowing is not a failure. It’s an invitation to listen, ask questions, and wonder like a med student again”. But this is also “when your curiosity becomes most powerful” (p. 19, [115]). What we know when we graduate is not the same as what we know when we retire, for the simple reason that medicine is always evolving and new treatments and discoveries are made. But also because new diseases, like Ebola, COVID-19, and long COVID, appear [116,117,118].

#### 4.7.2. Medical Gaslighting

However, blaming patients and simply claiming that every hoof step is a zebra in those cases is much easier than acknowledging that we (doctors) do not know everything. When doctors mistakenly conclude that a person’s symptoms are ‘all in their minds,’ they not only delay a correct physical diagnosis, but they also often subject patients to the wrong treatments and the potential side effects of those. Moreover, many patients have to mentally prepare themselves whenever they go to see a doctor, to be disbelieved and to be traumatized by the people who are supposed to help them [112]. Their suffering is often dismissed because they look too well and they are too functional by appearances to be able to be having problems from an invisible, often chronic disease [119]. Many of these patients turn out to have a (semi) rare disease. But the dismissive attitude of doctors can lead to years of unnecessary suffering before a patient finally receives the correct diagnosis [120]. Similar things happen to other diseases that many doctors do not believe in, like chronic and long Lyme, fibromyalgia, and now also long COVID. Consequently, medical gaslighting often results in delayed diagnoses, psychological distress, and poorer patient outcomes [121]. Moreover, a review that looked into gaslighting in gastrointestinal practices found that patients characterized by ‘psychosomatic’ symptoms and those with multiple symptoms are often deemed to be difficult or heart-sink patients. Patients with psychosomatic gastrointestinal diseases are labeled as having FGID, and many doctors believe that functional symptoms are all in the person’s mind. This is based on the assumption that “if modern medical testing cannot identify the root cause, then the root cause must be psychiatric” (p. 4, [122]). Physicians easily become frustrated when working with these patients because they do not want to waste their time with patients who, in their opinion, are not (physically) ill. One of the main culprits for this is the biopsychosocial model, which has claimed for decades that there is no underlying disease in ME/CFS because many doctors believe that if tests are normal, then there is no biological component to the disease, and then by definition it must be psychosocial, psychological, or psychosomatic [122].

According to Alfandre, in an article entitled *Medical gaslighting: when patients believe they are not taken seriously*, “it is time to call medical gaslighting what it likely is: patient dissatisfaction with their health care” (p. 1, [123]), while some others claim that a more appropriate term would be medical miscommunication instead of medical gaslighting [122]. However, as most patients know who have been on the receiving end of medical gaslighting, this is not caused by a form of miscommunication, a belief of not being taken seriously, or a form of dissatisfaction with their healthcare. Instead, it is down to doctors who do not believe that patients can have debilitating diseases if (routine) tests are normal and consciously or subconsciously think that they can, then behave towards those patients in a very derogatory, dismissive, but often also aggressive and hostile way. An example of dismissive comments by doctors is telling patients that “I feel tired all the time, too” (p. 30, [1]).

Patients often perceive this as doctors feeling superior to them and looking down on them. According to Sorrick [110], medical gaslighting is not the product of a few bad doctors, but it is a problem that is structurally embedded in medicine. In an article about medical gaslighting of long COVID patients, this is called a form of structural iatrogenesis by Lokugamage et al. [124].

Many doctors already have a bad opinion of a patient before the appointment has even started because, in the eyes of those doctors, they have difficult or frustrating diseases like POTS, fibromyalgia, chronic regional pain syndrome, or ME/CFS. Many doctors view these patients as hysterical hypochondriacs [125].

For patients with diseases like ME/CFS, this is made worse by claims from doctors that they are after secondary gains [126,127,128], by which those doctors mean that patients are after benefits without ever providing any evidence for that. The basis of medical gaslighting of patients with these diseases seems to be that doctors do not understand that absence of evidence is no evidence of absence. But also that if you do not do the right test, then you will not get the right answer. Or alternatively, if the right test has not been invented yet, then you will not get the right answer either. As concluded by McKay et al., just “because there is no diagnostic test or treatment, then CFS/ME must be a psychiatric condition, [is] a bizarre and erroneous conclusion” (p. 888, [129]).

#### 4.7.3. Healthcare Trauma

Another word for clinician-associated traumatization, according to Niederman, is “healthcare trauma: a sense of desperation, hypervigilance, and emotional wounding from being dismissed or covertly abandoned. Even as a veterinarian by training, I was unprepared for the vulnerability of being a patient. You don’t just fight a disease-you fight to be believed” (p. 19, [115]).

Disturbingly enough, in these sorts of cases, doctors also behave punitively towards patients, proactively undertaking actions to prevent their patients from pursuing the care or treatments they need [130]. The consequence of all this is that a person is no longer regarded and treated as a being of equal worth. When patients speak back, they often face more aggression and stigmatization [131,132]. Medical gaslighting often tries to ascribe symptoms to other causes, such as psychological issues or a patient’s need for attention. It is important to note that medical gaslighting, contrary to normal gaslighting, is usually not performed purposefully. Symptom invalidation or making a patient second-guess themselves is one of the hallmarks of medical gaslighting. By doing so, medical professionals are creating doubt in the patient’s mind that their symptoms might not be real. The consequence of this might be that patients do not seek additional medical attention, letting potentially harmful symptoms go untreated [110,125,130,133,134].

One has to ask the question, though, how far has a healthcare system gone astray when patients need therapy to seek necessary and often urgent psychological treatment to recover from encounters with medical professionals and harm inflicted on them by those medical professionals? Even more so because these patients know that the next time they go and see a medical professional, that trauma will be made bigger. Will all those medical professionals apologize (and more) to those patients who, after years of being incorrectly diagnosed and dismissed as having a psychosomatic disorder, have finally received the right or correct diagnosis because they were lucky to finally encounter a knowledgeable doctor who behaved in a professional instead of a gaslighting and dismissive way?

### 4.8. Neurocognitive Impairment

Neurocognitive impairment, which is often referred to as cognitive dysfunction or brain fog, is one of the four key diagnostic symptoms of ME/CFS according to the NICE ME/CFS guideline [2]. Many patients view this as one of the most disabling symptoms of the disease [135], which can have a marked impact on the level of disability and the quality of life of patients [136]. The Montreal Cognitive Assessment (MoCA) is a brief screening tool that can be used for assessing mild cognitive impairment [137].

#### 4.8.1. Patient Surveys

The British ME Association (*n* = 450) found that 88% of respondents with ME/CFS suffer from brain fog or cognitive difficulties [138]. According to research by de Becker et al. (*n* = 2703) [139], 93% of ME/CFS patients were suffering from attention deficit, 85% from memory disturbances, 75% had difficulty finding the right words, and 71% suffered from speech difficulties. Another survey about cognitive dysfunction from the British ME Association (*n* = 785), found that in 61% of patients, the severity of their cognitive dysfunction varies, whereas in 38% of respondents, cognitive difficulties are constantly there without varying. Additionally, in 16% of all respondents, which equates to 42% (126 ÷ 300) of those with constant cognitive dysfunction, their brain fog is very disabling [140].

#### 4.8.2. Common Other Symptoms

Cognitive dysfunction often leads to difficulty with information processing, slowed thought and speech, and impaired concentration. Patients might suffer from short-term memory loss, have poor working memory, or have difficulty remembering what they want to say. Other common problems of cognitive dysfunction are difficulty in speaking or mixing words up, whereby patients are aware of the mistakes they make, but they are unable to prevent them [140,141].

#### 4.8.3. Multitasking

Cognitive overload may be evident as slowed information processing speed, particularly on complex tasks requiring sustained attention or when two tasks are performed simultaneously, making multitasking very difficult or impossible [142,143]. As a consequence of the cognitive impairment, patients might have problems with talking to others, making telephone calls, reading, using a laptop or computer, watching TV, or performing a cognitive task that before they fell ill was no problem [68,140,141]. Severe cognitive dysfunction, which is often accompanied by irritation of the brain, in combination with hypersensitivity to sound, might mean that even talking to a very severely affected patient for a few seconds may result in further deterioration.

#### 4.8.4. Underlying Mechanism

The exact underlying mechanism for cognitive dysfunction is unclear, yet impairments in cognitive performance are associated with autonomic dysfunction, neuroinflammation, reduced cerebral perfusion, and elevated lactate levels in the liquor, which are consistent with the presence of energetic dysfunction [1,2,34,35,144,145,146,147,148,149,150,151,152]. For example, spectral analysis of EEG data produces a pattern that distinguishes patients with ME/CFS from both healthy controls and patients suffering from major depression. Also, fMRI identified unusual brain responses following cognitive, visual, and auditory challenges [153]. Neuroimaging studies of ME/CFS have also frequently observed additional brain area recruitment during cognitive tasks and abnormalities in the brain stem, which are not seen in healthy controls [154].

#### 4.8.5. Pain and Mood Disturbances

According to a systematic review [155], fatigue does not impact cognitive symptoms in ME/CFS or in multiple sclerosis. However, mood disturbances caused by depression can impact cognitive abilities, whereby the most affected domains are executive functions, attention, verbal working memory, and processing speed. The same systematic review noted that pain has a similar impact on cognitive abilities as depression, and when pain becomes too severe, patients cannot concentrate on anything anymore. However, the review also noted that talking problems in ME/CFS “cannot be reduced to the cognitive effects of depression and chronic pain” (p. 12, [155]), and the authors concluded that mood or pain can mediate cognitive dysfunction, but they do not cause it. Something similar was noted by Robinson et al. when they concluded that a comorbid major depressive disorder in itself is not responsible for a reduction in cognitive performance in ME/CFS [151]. Constant et al. not only concluded that there is an objective impairment in attention and memory in ME/CFS but also that cognitive impairment in patients with a major depressive disorder is strongly correlated with depression and fatigue, whereas there was a weaker correlation between cognition and depression and no correlation with fatigue in ME/CFS [156].

#### 4.8.6. Excluding Other Conditions

It is important to exclude other conditions that could be responsible for the cognitive dysfunction, like hypothyroidism, vitamin B12 deficiency, going through the menopause, drugs like sedatives, tranquilizers, anticholinergic drugs, and glucocorticoids, and undiagnosed or poorly regulated diabetes mellitus [140,157,158,159,160]. As well as different forms of dementia, mental health problems like depression and anxiety, brain tumors, stroke, alcoholism, kidney failure, and neurological diseases like Parkinson’s and multiple sclerosis [157].

#### 4.8.7. Treatment for Neurocognitive Impairment

There are no effective treatments for cognitive impairment caused by ME/CFS, which, just like physical impairment, can be worsened by exertion. Therefore, pacing of mental activities, just like patients do for physical activities, is an important strategy for patients to try to avoid further deterioration of cognitive function. An important strategy for carers and others when dealing with patients with severe cognitive dysfunction, to try and prevent further deterioration of their cognitive function, is keeping conversations *short, sweet, and simple* to make it easier for the brain to process the information presented.

### 4.9. Treatment for ME/CFS

For many years, guidelines all over the world have recommended CGT and GET as safe and effective treatments for ME/CFS, even though patients have been saying since the early 1990s that those treatments were neither effective nor safe. The IOM concluded in 2015 that there is no effective treatment [1], and a number of re-analyses of CGT and GET studies have highlighted the many problems with those studies as well as the absence of objective improvement and the negative effect of both treatments on work and disability status [161,162,163,164,165,166,167,168,169,170,171,172,173,174,175,176]. The conclusion by NICE in 2021 that both treatments do not lead to improvement or recovery confirmed the conclusion by the IOM that there is no cure and a dearth of effective treatments [1,2].

There are no FDA-approved treatments for ME/CFS itself, but comorbid conditions can often be treated successfully. In addition, symptoms such as pain, sleep difficulties, and autonomic problems can sometimes be addressed with medication. A common additional problem of ME/CFS is medication intolerance at regular doses; treatment strategies for symptoms or additional problems should generally follow the principle of *‘start low and go slow*’. Stimulant medications like methylphenidate or modafinil might help with fatigue and brain fog, but risk worsening hyperadrenergic symptoms and PEM, and therefore should be used carefully [61]. The mainstay of ‘treatment’ for ME/CFS is pacing, which is an energy management strategy [2]. Moreover, there are some off-label treatments that we will discuss in a minute.

#### 4.9.1. Pacing

The only remaining option for patients is pacing, which has been advocated by them for a long time. Pacing is not a therapy or an effective treatment, nor is it curative. It is simply a self-management strategy whereby patients try to stay within their limits, be they cognitive or physical, and only do around 70% of what they can do. Not only to try and reduce the risk of developing PEM but also to try and reduce the risk of a flare-up or a relapse in an effort to try and prevent further deterioration of the disease [2,177,178].

#### 4.9.2. Pyridostigmine (Mestinon)

One small non-randomized study (*n* = 20) without a control group that used hand grip strength testing showed that there was an immediate effect on muscle strength and orthostatic function after administering 30 mg of the acetylcholinesterase inhibitor pyridostigmine [179]. However, it is impossible to say anything with certainty about causality because of the design of the study.

#### 4.9.3. Low-Dose Naltrexone

Naltrexone is a safe synthetic anti-opioid that is used for the treatment of alcoholism and opioid addiction at 50 mg. At lower doses, 3–4.5 mg, it appears to work as an immune modulator. The mechanism of possible action for low-dose naltrexone (LDN) in ME/CFS is unknown. Bolton et al. [180] described three different cases who all continue to take LDN but with differing success, from a reduction in some symptoms only to life-changing differences. Treatment doses ranged from 4 to 12 mg.

They also note that internet reports suggest that side effects in the form of increased fatigue and headaches can be troublesome initially for patients. In one of the three patients, the frequent episodes of abdominal pain subsided when taking 9 mg, and she was able to start eating a normal diet, including wheat and dairy [180].

A small study involving nine patients and nine healthy controls found that ME/CFS patients taking LDN (3.0–5.0 mg/day) have restored TRPM3-like ionic currents in NK cells, which supports the hypothesis that LDN may have potential as a treatment for ME/CFS [181]. On the Internet, there are accounts from patients who have found LDN helpful and others who have not.

#### 4.9.4. Low-Dose Rapamycin

Ruan et al. [182] concluded that rapamycin led to recovery in fatigue, PEM, and orthostatic intolerance in 74.3% of patients, 30 days after starting low-dose rapamycin (6 mg/week). According to the study, it was well tolerated, and there were no serious side effects. However, 23 of the 109 patients who enrolled in the study refused to start treatment after the baseline assessments, only 55 completed the 60-day assessment, and only 40 completed the full 90-day study protocol. This raises doubt about the conclusion of the study that “the weekly low-dose rapamycin regimen was well tolerated, with minimal adverse events (AEs) reported” (p. 8, [182]) because normally patients who do not find a treatment effective or in whom it may even be harmful, are the ones who drop out [183].

As the authors rightly concluded, there were some major limitations of their study. These included the absence of a control group and the reliance on self-report measures. At the same time, they are addressing those issues by having started a placebo-controlled study that uses the actometer and VO2max as objective outcome measures to provide more reliable information on whether low-dose rapamycin might be an effective treatment for ME/CFS or not.

#### 4.9.5. Immunoglobulins

In the past, it was said that the four trials that studied the efficacy of intravenous immunoglobulin G (IV IgG) for ME/CFS showed mixed results. However, a reanalysis of those studies by Brownlie et al. showed a different picture, and they concluded that the interpretation of the literature regarding the issue had been faulty [184]. They found that patients in those studies either did not respond at all or the treatment was highly effective. Interestingly enough, it was especially effective for the severely affected. In the article, they also use five cases of adolescents because one of their authors is the leading pediatric ME/CFS specialist in the UK. Three of those were 13-year-old girls, and one was a 19-year-old young woman with very to extremely severe ME/CFS, and all four of them made a complete recovery over a timeframe of two years. The same applied to a boy who had moderate ME/CFS, who was the only one of these five patients who was able to come to the hospital for treatment with IV IgG. The other four were treated with either intramuscular or subcutaneous IgG as an alternative to avoid the necessity of traveling to the hospital. Brownlie et al. therefore concluded “that immunoglobulin is a potentially curative treatment for a proportion of patients” “with severe and well-characterized ME/CFS” and “that predictors of response lie in indicators of abnormal cell-mediated immunity” (p. 1, 15, [184]). By that they mean, for example, “CD4 (helper T) and CD8 (suppressor/cytotoxic T) lymphocyte count”, “absolute count of the T-cell subsets”, “CD56 (natural killer)”, and “cutaneous delayed-type hypersensitivity (DTH)” (p. 18, [184]).

As far as adverse effects are concerned, treatment with immunoglobulin was associated with mild, transient, and self-limiting adverse effects, most notably headaches.

Brownlie et al. finish by concluding that “clinicians would be justified in offering a course of IgG to selected ME/CFS patients at the more severe end of the spectrum” (p. 1, [184]). They also conclude that the fact that the majority of patients in the studies had a viral onset of their ME/CFS suggests that IgG might be a treatment option for patients with long COVID. A retrospective matched small case–control study in long COVID with 10 patients in the IgG treatment group, confirmed that [185]. What is now needed are large and properly designed placebo-controlled studies that use objective primary outcome measures to see if IgG is really a treatment option and an effective treatment for the more severely affected ME/CFS and for long COVID patients or not.

#### 4.9.6. Caffeine

The US ME/CFS Clinician Coalition recommends caffeine as a treatment for cognitive dysfunction [186]. However, one has to be careful with taking caffeine as a supplement or drinking it in coffee or energy drinks, because it means that patients will not feel their boundaries very well. As a consequence, it is much easier to go over them and cause PEM, a flare-up, or a relapse. For the same reason, one has to be even more careful, or extremely careful, with taking even very small amounts of caffeine during PEM, flare-ups, or relapses.

### 4.10. Access to Medical Services and Additional Problems

The severely ill are homebound and bedbound, and they are generally too ill to travel to outpatient appointments or to attend doctors surgeries. Consequently, they require home visits, yet a study by McDermott et al. [187] found that in many areas of the UK, this is not provided even though it was recommended by NICE. Some of the areas were willing to see patients from other areas, but the authors noted that traveling presents a major problem for the severely affected, therefore out-of-area referrals may be of limited use for this patient group unless inpatient stay is offered. Traveling might be too exhausting, and it might also expose patients to too many sensory inputs, which can lead to PEM and severe flare-ups and relapses. Moreover, the same applies to inpatient stays, as most hospitals and wards are not set up to deal with the severely ill [188].

Many people with severe ME/CFS commonly receive no care from healthcare professionals. Paradoxically enough, the more severely ill ME/CFS patients are, the more neglected they are by the medical profession and the less likely it is that they will receive any form of healthcare [189]. This is also known as the paradox of healthcare, which is often referred to as the inverse care law, a term invented by British GP Hart in the early 1970s, whereby the need for medical care is inversely related to the availability of it [190]. Put differently, the more severely ill patients are, the less help they receive from the medical profession. People with ME/CFS often make an enormous effort to prepare for a visit by a doctor, yet the resulting post-visit PEM and exhaustion are never witnessed by visiting doctors but can be a major deterrent for patients to ask for medical help, as the PEM caused by such efforts may continue for hours, days, or even weeks. Or, depending on the length of the visit and the attitude of the doctor, it might also lead to relapses.

It is advisable that healthcare professionals should avoid smoking or the use of perfumes or fragranced lotions, as sensitivities to chemicals or odors are commonly seen in the severely affected. If doctors have symptoms of infections, then they should avoid visits, as some patients are more susceptible to infections, and in many cases it can lead to worsening of the disease.

People with severe ME/CFS may only be able to stand very shortly, if at all, and signs of weakness on physical examination may be obvious. Handgrip tests often show reduced strength in the severely affected. It is important not to rely on testing only once but to repeat these tests a number of times to show the rapid decline when repeated, even when initially, power seemed normal or fairly normal.

Cognitive dysfunction is a common symptom, as we have seen earlier, just like irritation of the brain. If the latter is the case, then it is important to keep conversations *short, sweet, and simple*. Do not talk loudly or make unexpected noise because many severely affected patients are extremely sensitive to even the most normal sounds. Do not use your mobile phone around them, and make sure that it cannot ring, vibrate, or make other sounds to prevent further irritation of the brain and to try and prevent further deterioration [27]. Patients can be so ill that they cannot tolerate the presence of other people in their room. Therefore, before you go into their room to see them, check with their family if they can tolerate your presence, even if it is incredibly short. Do not be offended if they want to keep the visit or conversation very short to prevent further irritation of their brain. That has nothing to do with the person in question, but all with the irritation of the brain. Even a visit or conversation of less than a minute can be very long. As a matter of fact, these patients have to deal with a paradox. They are not only fighting all sorts of horrible symptoms and a debilitating disease, but also boredom and loneliness if their brains are too irritated to do anything other than stare at the ceiling, while lying in dark and quiet rooms. In those sorts of cases, they long for contact with others, yet their brains are too irritated to engage in interactions with others, leading to the paradox of longing for contact but being unable to do so [27].

### 4.11. Caring for Someone with Very Severe or Extremely Severe ME/CFS

Since patients with severe ME/CFS should be left in peace as much as possible, active care—apart from emergencies—usually consists of a series of interventions that are as short as possible and spread out throughout the day. When ME/CFS patients are subjected to stress beyond their tolerance threshold, their bodies react with deterioration of their condition, which is known as PEM or a crash [27]. In severely affected individuals, the stress tolerance threshold is often so low that crashes can be triggered even by essential activities (such as eating or turning over in bed). In such cases, the affected individuals cannot fully recover from a crash because they already suffer the next one; this is also known as “rolling PEM”. Even in this condition, more severe crashes with further functional loss are possible and must be avoided at all costs. The central goal of caring for someone with very severe ME/CFS is therefore to prevent crashes and the associated deterioration of their condition. Another goal is to try to avoid malnutrition. One of the ways of trying to achieve that is by having a daily routine to ensure adequate food and fluid intake. Feeding difficulties can arise due to several factors that often occur simultaneously. Extreme exhaustion and muscle weakness can mean that eating is very strenuous because chewing and even swallowing can be (too) strenuous. Many patients with very severe ME/CFS also have food intolerances and allergies, often due to mast cell activation syndrome (MCAS). Additionally, the aforementioned autonomic dysfunction of the esophagus, stomach, and/or the intestines can make food intake considerably more difficult or even impossible, which can lead to life-threatening malnutrition, as is the case with Paula, but which is often not recognized and acknowledged by the hospital [26].

A schedule of 4–5 smaller meals is often helpful, but must be individually tailored, especially if the person is unable to eat frequently. If the person is able to eat independently, or if the presence of others is more burdensome than the physical exertion, simple aids such as lightweight plastic dishes, plastic cups with straws, and non-slip plastic trays can be helpful. If sitting up is not possible, but the patient can still turn on their side, then some patients can still eat independently if a plate is placed on their bed with small pieces of food, which they then eat directly with their mouth from the plate. For many patients, pureed food or soups are the most easily tolerated, especially if chewing is too strenuous or too noisy. If all that fails or eating is simply too strenuous to do, then high-calorie liquid nutrition can be helpful. Tube feeding may be necessary in cases of severe intolerances or problems with chewing and/or swallowing [26,27]. As noted by NICE, some very severely affected patients might not be able to swallow at all [2]. This should be considered early instead of late to try to prevent dangerous weight loss. NG tubes, which can be inserted at home, often provide significant relief because patients no longer have to worry about feeding. But if that does not work, then patients have to go to the hospital for other feeding tubes (PEG, PEG-J) or even to be treated with PN. The additional problem, as we have seen in the case of Paula, is that Dutch hospitals refuse to treat severe malnutrition in ME/CFS with PN, even if that is the only option.

Other instructions and helpful tips for caring for patients with very severe or extremely severe ME/CFS can be found in the nursing instructions for seriously and critically ill ME/CFS patients (in German: Pflegeanleitung für schwer und schwerstkranke ME/CFS Patient:innen) from the University of Vienna, which was developed together with the Austrian ME Association [27].

### 4.12. Suicide Risk and Depression

According to a Swiss survey, 39.3% of respondents had suicidal thoughts at some stage, 14.8% were currently suffering from a secondary depression, and 78% of those had never suffered from it before falling ill [162]. The three main factors contributing to suicidal thoughts were (i) being told the disease was only psychosomatic (89.5%), (ii) being at the end of one’s strength (80.7%), and (iii) not feeling understood by others (80.7%). At the same time, they found that the occurrence of mild depression or anxiety was often associated with a specific notable event, such as an accident, the loss of a loved one, unemployment, or a relationship break-up/divorce. Lack of awareness and disease knowledge, as well as the absence of disease-specific markers, are known to increase stigmatization in society [191]. This was also observed in long COVID, Ehlers–Danlos, multiple sclerosis, and Lyme disease [81,117,130,132,192]. König et al. finish the article by concluding that “this study highlighted the profound impact of the added mental strain resulting from ME/CFS, primarily stemming from widespread stigmatization, disbelief, a lack of understanding, inadequate medical support, and limited disease awareness” (p. 12, [191]).

Even though there is no effective treatment, treating these patients with empathy, compassion, dignity, and respect and showing them that you are there for them as a doctor if they need medical attention or help, is not only reassuring, but it also removes a lot of stress related to medical encounters [189]. At the same time, it might help to reduce the risk of suicide, which is similar to other chronic illnesses. Suicide is much more common than in the general population and occurs at a younger average age, too. Risk factors for this are not only the stigma caused by the belittling term chronic fatigue syndrome, the continual presence of pain, the severe irritation of the brain, and the significantly decreased functionality, but also unsupportive and often incredibly hostile interactions with medical professionals [193,194,195,196]. A good example of this is a remark by one of the therapists in the FINE trial, the only study that investigated the efficacy of CGT and GET in the severely ill. That therapist said the following. “The bastards don’t want to get better” (p. 7, [197]).

### 4.13. Prognosis

According to an influential systematic review, only 5% of patients recover, while a larger percentage of patients have some form of improvement [198]. Severely ill patients tend to have poor prognoses, whether children or adults. Improvement in the condition of the severely ill is even less likely [189]. According to the aforementioned survey of the European ME Alliance [85], only 0.2% of severely ill patients recover. “When patients aren’t getting better, or are even getting worse, don’t assume there’s nothing left to do. Because you can still offer something rare: a steady presence in uncertainty” (p. 20, [115]).

According to a systematic review by Joyce et al. [199], consistently reported risk factors for poor prognosis include older age and a more chronic illness. A study of 1104 patients by Pheby et al. found that 37.9% of severe cases reported a family history of ME/CFS, compared with only 17.1% of mild cases. Also, there was a strong association between having a mother with ME/CFS and developing severe disease, less strong associations with having a sibling or child with ME/CFS, and no association at all with having a father with the illness [200].

Pheby et al. also concluded that “the strong association with having a mother with ME/CFS, but lack of association with having a father with the condition, is consistent with ME/CFS being associated with disturbed mitochondrial function. Mitochondrial DNA is of course entirely of maternal origin” (p. 68, [200]). The study by Pheby et al. also found that early management is implicated in the prognosis and development of severe disease. Yet personality is not a prognostic factor. An extensive review of the literature found that the prognosis in terms of returning to work is poorer for older people and those who were more ill at the beginning of their illness. Moreover, it is unlikely that patients will be able to return to work if they have been off sick for more than 2 to 3 years [162]. Patients who have a period of enforced rest in the initial stages of their illness tend to have the best prognosis [71,162].

Finally, as concluded by Dowsett et al., “ME predominantly affects the most socially and economically active section of society. Misinterpretation of this common illness as psychogenic delays the early recognition mandatory for modification of lifestyle which may avoid progression to chronic disability” (p. 530, [3]). Damaging treatment (exercise) negatively affects the prognosis and makes it more likely that patients will develop severe ME/CFS [71,162,201].

## 5. Discussion and Conclusions

### 5.1. Severe ME/CFS

In this article, we have used the case of a woman with life-threatening malnutrition as a consequence of a number of diseases, including severe ME/CFS, to illustrate and discuss the problems this disease can cause and the impact it can have. We have reviewed the evidence and the literature regarding severe ME/CFS, and we have found that there are no simple diagnostic blood tests, biomarkers, or evidence-based, effective pharmacological treatments. There is, however, a diagnostic test in the form of 2-day cardiopulmonary exercise testing (2-day CPET). The consensus, however, is that, because of the potential harmfulness of the test, it should only be used in research settings or when there are disability disputes, as many insurance doctors and other doctors still do not believe in the disease based on the fact that patients often look well, routine testing is normal, and medical journals and textbooks have been backing and promoting the psychosomatic view of the disease for more than 30 years.

According to some, for example, Dutch sport physician Janssen, the way to limit the potential harmfulness of the test is by performing a submaximal test on two consecutive days supervised by exercise physiologists, cardiologists, or sport physicians, who are knowledgeable about PEM [202]. It is, however, important to stress that at present ME/CFS is a clinical diagnosis based almost entirely on a carefully taken history and using diagnostic criteria like the Canadian consensus criteria or the international consensus criteria [68,141].

The exact etiology of the disease is still unknown, even though in most people it follows or is triggered by a viral infection, and even though it can impact anyone, it is particularly prevalent in women, just like many autoimmune diseases [203]. According to a study by Falk Hvidberg et al., the health-related quality of life in ME/CFS is lower than in many major medical conditions, including acute myocardial infarction, cerebral thrombosis, end-stage renal disease, congestive heart failure, angina, rheumatoid arthritis, multiple sclerosis (MS), lung cancer, stroke, or ischemic heart disease [79]. Something similar is now also seen because of the COVID-19 epidemic, which can lead to long COVID, which in almost 90% of cases is similar or the same as ME/CFS [204].

### 5.2. The NIH and Altered Effort Preference

A recent National Institutes of Health (NIH) study by Walitt et al. [205] concluded that post-infectious ME/CFS is a disorder defined by altered effort preference, leading to activity avoidance and subsequent deconditioning. Many doctors would see this as confirmation of their skepticism about the existence of this disease, but also that ME/CFS is a psychosomatic disorder. However, this small study, which compared only 17 ME/CFS patients to healthy controls, illustrates the problem of using one day instead of a 2-day CPET. As noted by exercise physiologists in 2013 and the Institute of Medicine (IOM) in 2015, a second test is often necessary to document the atypical recovery response in ME/CFS [1,206]. If one does not use a second test, then it can be impossible to distinguish the problems encountered by ME/CFS patients with exercising from deconditioning. The NIH study also highlights the lack of knowledge by doctors about PEM, as the study characterizes this as “feelings of discomfort” associated with exertion (p. 2, [205]) and the description of patients’ “conscious and subconscious” behavioral “alterations” as attempts “to avoid discomfort” (p. 10, [205]). Whereas we have seen earlier, PEM is not discomfort and trying to avoid PEM is not an abnormal response. In a similar way that if you have a badly broken ankle, for example, then most people will avoid running or other forms of exercise that require the normal use of this ankle. This is not an abnormal response or a form of altered effort preference. Instead, it is not only the sensible thing to do to prevent further damage, but the injury or the disease (ME/CFS) itself also prevents one from doing it.

### 5.3. What Is PEM?

As noted before, many health professionals think that post-exertional fatigue, i.e., tiredness after exercise, is PEM, even though tiredness after exercise is simply a normal physiological response to exercise. Some also claim that worsening of symptoms after exercise is PEM. Yet something similar is seen if people are injured and they return to sports too quickly or do too much too quickly. PEM, however, is something different. PEM may occur immediately after activity or be delayed by hours or days [68], and the following four elements are compulsory for a diagnosis of PEM:

- a disproportional worsening of symptoms,
- following trivial physical or mental exertion,
- with loss of strength and/or loss of function,
- and an abnormally delayed recovery [70].

### 5.4. A Physical Disease

The finding by Walitt et al. in their supplementary data (45107_MOESM1_ESM) that patients switch to the anaerobic metabolism earlier than healthy controls which according to them, “demonstrates [the] less efficient oxidative respiration in PI-ME/CFS participants” (p. 13, [205]) and confirms that ME/CFS is a physical disease and not a psychosomatic one. The finding that exposing muscle cells from healthy people to serum from ME/CFS patients, which causes muscle weakness as well as severe muscular and mitochondrial deterioration, confirmed that as well [207]. This is in line with the conclusion from a recently published study that analyzed CGT and GET studies to show that those studies have proven that ME/CFS is a physical disease [208]. It is also in line with the conclusions by the prestigious American Institute of Medicine in 2015 and the British NICE Institute in 2021, based on an extensive review of the literature, that ME/CFS is a debilitating “complex, chronic medical condition affecting multiple body systems” (p. 9, [2]) characterized by post-exertional malaise (PEM) and at least a 50% reduction in functioning, in combination with a range of symptoms which often include cognitive dysfunction, sleep reversal or other sleeping problems, muscle pain and muscle weakness, hypersensitivity to light and/or sound, tinnitus and dizziness, and headaches and migraines [1,2].

### 5.5. Neurocognitive Impairment

Neurocognitive impairment, which is often referred to as cognitive dysfunction or brain fog, is one of the most disabling symptoms of the disease. It is also one of the four key diagnostic symptoms of ME/CFS according to the NICE ME/CFS guideline [2]. Even though patients might not suffer from it in the initial stages of the disease or when they have a very mild form of ME/CFS. Cognitive dysfunction can present in many different ways, including, for example, difficulty with information processing, slowed thought and speech, impaired concentration, memory disturbances, and difficulty finding the right words. Cognitive dysfunction might lead to problems with, for example, talking and communicating with others, reading, using the computer or the Internet, multitasking, or making telephone calls. In its most extreme form, it is impossible to do all that and more. Hypersensitivity to light and sound can have a profound negative influence on the level of cognitive dysfunction.

The precise underlying mechanism of cognitive dysfunction is unknown, although research suggests that autonomic dysfunction, neuroinflammation, reduced cerebral perfusion, and mitochondrial dysfunction might lie at the base of it. There are no effective treatments for it. Therefore, pacing of mental activities, just like patients do for physical activities, is an important strategy for patients to try to avoid further deterioration of cognitive function. A commonly used strategy in dealing with very or extremely affected patients is keeping conversations *short, sweet and simple* to make it easier for the brain of the affected patient to process the information presented.

### 5.6. Medical Gaslighting

Most doctors do not realize that the fact that tests are normal is no proof that something is psychiatric or psychosomatic, nor do they realize that there is never any proof that something is psychosomatic for the simple reason that such proof does not exist. As we have also seen in this review, the consequence of that is that many patients with diseases like ME/CFS, but also with, for example, chronic or long Lyme, fibromyalgia, hypermobile Ehlers–Danlos syndrome, and other invisible diseases, are often subject to medical gaslighting, which in many cases leads to healthcare induced trauma or traumatizing of patients [110,111,112,113,114,115,116,117,118,119,121,122,123,124,125,130,131,132]. Ironically enough, patients who only had a physical disease before they were seen by the medical profession, then suddenly also need to see mental health professionals to deal with the healthcare-induced trauma, which is a form of avoidable iatrogenic harm [209].

Another typical example of medical gaslighting is changing a physical diagnosis into a psychosomatic one. In the case of the Dutch patient we discussed in this article, the doctors changed the severe bowel problems and life-threatening malnutrition, caused by three physical diagnoses, into problems caused by a psychosomatic one, i.e., a functional disorder, without providing any proof for that. Or alternatively, they simply ignored the problems those diseases can cause and then claimed that the problems are caused by a functional disorder. Why doctors and the medical profession think that medical gaslighting is normal and acceptable is unclear.

### 5.7. Categories of Severity of ME/CFS

It is generally assumed that there are four categories of severity of ME/CFS: mild, moderate, severe, and very severe. The severe and very severe are often overlooked in studies. This is even more the case for the fifth category, the extremely severely ill patients, which is usually not only overseen but also never mentioned. These patients are also referred to as the living dead, as they are as sick as patients with late-stage AIDS, yet they have to live like that for years or decades [87,210]. A photography project entitled *The Living Death* has focused on photographing this form of life and has won a number of photography awards for it [211]. For example, the updated NICE ME/CFS guideline does not mention this category, just as it is not mentioned in other guidelines [1,2,212,213]. The University of Vienna, however, recently published an extensive manual on how to care for the most severely affected patients [27], and earlier we highlighted and discussed a number of items from that manual. Freitag et al. reported in 2021 that autoantibodies against G-protein-coupled receptors correlate with symptom severity, autonomic dysfunction, and disability in ME/CFS [214].

### 5.8. Treatment and Pacing

In this article, we have seen that pacing is the only thing patients can do to try to prevent deterioration in the absence of effective treatments for ME/CFS. It is important to note that pacing is only an energy self-management strategy. Pacing is not a therapy or an effective treatment [2,177,178].

We have also discussed a number of treatment strategies like low-dose naltrexone (LDN), low-dose Rapamycine (LDR), immunoglobulins, the acetylcholinesterase inhibitor pyridostigmine, and caffeine, for which there is limited or no evidence of efficacy [179,180,181,182,184,185,186]. For example, a large RCT of LDN for ME/CFS has not been done. The LDR and the pyridostigmine studies were non-randomized studies without a control group. On top of that, the LDR study (*n* = 109) had a very high dropout rate (63%, 69 ÷ 109), and the pyridostigmine study was small (*n* = 20) [179,182].

One should be careful with caffeine because it makes it more difficult for patients to notice their boundaries. This can be especially troublesome during PEM, a flare-up, or a relapse. A reanalysis of the four immunoglobulin studies from the past showed that especially severely affected patients might benefit from it [184]. However, what is needed are large, properly designed studies to come to reliable conclusions about the safety and efficacy of immunoglobulins, LDN, LDR and pyridostigmine. Such a LDN study (*n* = 160) has just finished. Its results will be published later in 2026 [215].

### 5.9. Prognosis

The prognosis for ME/CFS in general is bad, as only 5% will spontaneously recover, but the chances for that, if people have been ill for more than 2 to 3 years, are very slim [71,162,198]. The prognosis for the very or extremely severely ill to recover spontaneously is only 0.2% [85]. Stories on the Internet from patients who claim that they were severely ill with ME/CFS but then have recovered, for example, because of a positive attitude, or by standing in a circle and shouting, *I don’t do this disease anymore* or by simply doing the things they want to do, are nothing more than anecdotal ‘evidence’ [216,217]. In those cases, one always has to wonder about the correctness of the diagnosis, as highlighted by analyses of those articles [218,219]. Moreover, if a positive attitude would be the cure for this disease, then people who are (very) severely or extremely severely affected would not be ill in the first place.

Additionally, it is always important to wonder if the diagnosis is correct, not only in those cases of miraculous recovery but also in other cases, because research by three different teams of researchers has shown that in around 50% of cases, the diagnosis is incorrect [220,221,222].

### 5.10. Pushing Your Boundaries

If ignoring your limits and pushing through your boundaries has a positive effect on the disease, then by definition the diagnosis was wrong because if patients with a disease which is characterized by PEM do that, and the same applies for the estimated 75% of long COVID patients who suffer from that [223], then this will induce PEM, flare-ups and severe relapses. From that point of view, it is very worrying that there are many exercise studies being performed on long COVID patients, which do not investigate if and how many patients in their study population suffer from PEM. But secondly, many of those studies, which are often non-randomized studies without a control group, as found by a recent review [70], then go on to claim that exercise therapy has a positive effect on PEM, whereas in reality, it is very harmful and it will render many patients bedridden for life in the absence of effective pharmacological treatment. Moreover, in the absence of a control group, one cannot come to any forms of claims about causality [162]. We recently declined the invitation to review a non-randomized exercise study without a control group in ME/CFS that, according to the abstract, was making the same claims yet was suffering from the same problems. These claims also highlight the absence of knowledge about basic trial design and post-infectious diseases by doctors in general and about PEM in particular.

### 5.11. Suicide and Euthanasia

We have also highlighted the dismissive attitude of the medical profession towards diseases they do not understand. This attitude also plays an important role in the fact that many patients cannot, on one hand, stand their suffering anymore, and on the other hand, they cannot stand the dismissive and hostile attitude of the medical profession towards them anymore. The consequence of this, as we have seen a number of times recently in the Netherlands and in Germany [224,225,226,227], is that some patients end their own lives via suicide, and others go down the euthanasia route, which under certain circumstances is legal in, for example, the Netherlands.

### 5.12. Systematically Neglected

A study by psychologist La Cour highlighted the fact that even the very and extremely severely ill are often removed from the list of their family doctor [228]. Not because they have misbehaved, but because the doctor does not believe in their disease. But why would patients not deserve healthcare even if they would have a psychological or psychosomatic disease or disorder?

La Cour also concluded that “the most severely ill patients with ME/CFS and their caregivers must be characterised as a systematically neglected patient group not comparable to any other similarly ill group” (p. 1, [228]). We mention this because one of the things that a number of Dutch patients who have requested euthanasia have mentioned is that, for the first time, while being (very) severely ill with ME/CFS, they were treated with compassion, respect, and humanity by the medical profession. People who go down the euthanasia route are, in general, positive people who cannot stand the suffering anymore, yet they are not depressed. What they really want is to have effective treatment and to have some form of health and life back. But they are left without an alternative in the absence of effective treatments and the lack of medical curiosity to not only find out what the underlying disease process is, but also to provide these severely ill patients with effective pharmacological treatments. The consequence of that is that there is no end in sight to their suffering, which leads them to go down the euthanasia or suicide route.

### 5.13. Neglected Needs of Carers

Another consequence of medical gaslighting and psychologizing this disabling physical disease for decades is that the severe lack of knowledge about the current state of research into this disease means that many carers are not receiving any help from the medical profession. Instead, they need to figure out via trial and error what the best way is to help the very severely and extremely severely ill [228]. Without these carers, many thousands of people would have died, abandoned by the medical profession. The consequence of this is also that the carers will make mistakes because all they can do is learn by doing, but unfortunately, the margin for error in people who are extremely sensitive to light and sound, for example, is often nonexistent. Even the most minute sensory stimulus, which healthy people would not even notice, might or will have major repercussions for the already very fragile health of these severely ill patients, rendering them even more ill and even more dependent on their carers [27].

### 5.14. Accused of Child Abuse

As also concluded by psychologist La Cour, “in addition to the isolated caring conditions, several caregivers in this study had been suspected or accused of Münchausen by Proxy or incest. Through many years of essential caring, they have received neither recognition nor respect for the burdensome and time-consuming obligations otherwise left to society and public health care. The caregivers seem to be just as systematically neglected as the most severely ill ME patients themselves” (p. 9, [228]). As long ago as 2006, Colby already noted “that many affected children struggle for recognition of their needs, and are *bullied by medical and educational professionals* [italic by us]. Children should have time to recover sufficiently before returning to school; sustainable, energy efficient and often home-based education is important here to fulfil legal obligations” (p. 125, [229]). Dowsett and Colby concluded in 1997 that “of all the symptoms associated with ME/CFS, disturbance of cognitive function is the most disabling and long lasting” problem, interfering in a major way with the ability to go to school, do homework, or go to work (p. 5, [135]).

The Tymes Trust charity has helped and supported 121 families facing suspicion/investigation for child abuse, Münchausen by Proxy, or neglect, yet “none of these families has been found to be at fault” (p. 1, [44]). The only reason why parents and children were facing these allegations was the lack of knowledge about ME/CFS in general and the lack of knowledge about its severity by the doctors and social services that were involved, in particular. But going down this road by doctors and social services without any proof to back up their claims is another form of healthcare-induced traumatizing of patients, in this case, of children and adolescents, and their parents.

### 5.15. Going Forward

One of the ways forward to speed up the process of providing these severely ill patients with appropriate help and treatment is not only by spending more money on research and education of the medical profession, but also by performing more high-quality biomedical studies. It is important that these studies include the (more) severely affected patients, even though that will mean that researchers will need to do home visits using adequate methods that are sensitive enough because of the nature of the affected [83]. But there is also another reason to use this group of patients, because the more ill patients are, the less likely it is that they suffer from an alternative explanation for their fatiguing illness. Experienced ME/CFS clinicians will immediately recognize the very or extremely severely affected patients so that, in the absence of a diagnostic blood test or a biomarker, the chances of including patients in a study who do not have the disease under investigation are almost negligible.

#### Accelerating Research on ME/CFS

To be able to go forward and accelerate the knowledge about this disease, it is important to focus on a number of important issues. With the help of modern technology, including precision medicine and AI driven multi omics [78], it is important to find a biomarker that will help to more accurately diagnose the disease. This will also help to speed up the process of finding effective pharmacological treatments. A recent example of a study that used advanced technology was the aforementioned study that developed 3D in vitro skeletal muscle tissues to map the adaptations of muscle from healthy people to serum from ME/CFS patients over time. Short exposure (48 h) to patient serum led to an increased glycolysis and a significant reduction in the contractile strength of the muscle of a healthy person. Prolonged exposure (96–144 h) led to severe muscular and mitochondrial deterioration and caused muscle fragility and weakness [207]. This study by Mughal et al. [207] confirmed the finding from a 2015 paper that demonstrated that simple technology in the form of lactate testing after trivial exertion in a patient with severe ME, is still relevant and important [230]. It also showed that in that particular patient, there is also an increased glycolysis because the main cellular energy production process, the aerobic one, is severely impeded [230].

Repurposing medication that is already in use for other diseases might be one of the ways to find effective pharmacological treatments as quickly as possible. Investigating and elucidating the metabolic dysregulation, the pathophysiology of PEM and exertion intolerance, the neuro-inflammatory involvement, the potentially important T cell activity and natural killer cell activity, the role of circulating microRNAs, autoimmunity, for example, autoantibodies directed against EBV mimicking arginine-rich sequences in human proteins and the possible involvement of reactivation of herpes and other dormant viruses in the pathogenesis of ME/CFS, will be important ways to elucidate the underlying disease mechanism [78,231,232,233,234]. It is also important that patients and their representatives are involved in these studies so that outcome measures are used that are relevant to patients but also to society. The ultimate goal of patients, but also of their families and society, is not only to regain their health and independence, but also to be able to go back to work and be financially independent again.

## 6. Conclusions

This review highlights the disabling nature of ME/CFS in its most severe forms, the absence of a diagnostic test and effective treatments, and the lack of knowledge about this disease by medical professionals. We have also highlighted that in research settings and disability disputes, 2-day cardiopulmonary exercise testing can be used to diagnose and document the abnormal response to exercise. Yet this test cannot be used in routine medical care because of its potential harmfulnes. Biomedical research into this disease has been scarce and underfunded for decades, and research involving the most severely affected has hardly been performed at all. As a consequence of the COVID-19 pandemic, there is a sharp increase in the number of patients with post-infectious diseases. Many of these patients fulfill ME/CFS criteria. Dedicated, focused research involving the most severely affected is needed to gain further understanding of the underlying disease mechanism and to find effective pharmacological treatments to address the unmet medical needs of millions of people.

## Figures and Tables

**Table 1:** Spectrum of severity in ME/CFS.

Level of Severity	Description of Level of Functioning and Disease Severity
Mild	Mobile and able to self-care. Might be working or attending school, but often with accommodations and by reducing domestic and social activities. Patients have a substantial (50%) loss of function compared to before the onset of the disease.
Moderate	Reduced mobility and restricted activities of daily living. Requiring frequent rest periods and typically not working or attending school.
Severe	Mostly homebound. Limited activities of daily living (e.g., self-care, showering, dressing). Often with cognitive difficulties. May be wheelchair dependent.
Very Severe	Bedbound 24/7. Unable to care for themselves and dependent on care from others. They need help with personal hygiene and often with eating, and are very sensitive to sensory stimuli such as light, sound, touch, etc., usually with severe cognitive difficulties. Some people may not be able to swallow and may need to be tube-fed.
Extremely severe	Bedbound 24/7. Totally depended on others. Extreme sensory sensitivity to light, sound, touch, etc., accompanied by severe or extreme cognitive difficulties. Lying flat in silence and darkness to avoid deterioration. Many of them are malnourished and are tube-fed, incontinent, unable to communicate, intolerant to medications, and unable to move. Life may be reduced to basic survival, struggling moment by moment to breathe, eat, and drink, while enduring extreme pain. This form of ME/CFS is also referred to as the living death.

Sources: [2,10,13,27,30,35,41,43,44,57,61,68,71,83,84,85,86,87,88,89,90].

## Data Availability

Not applicable.
